# Anchoring the Late Devonian mass extinction in absolute time by integrating climatic controls and radio-isotopic dating

**DOI:** 10.1038/s41598-020-69097-6

**Published:** 2020-07-31

**Authors:** Anne-Christine Da Silva, Matthias Sinnesael, Philippe Claeys, Joshua H. F. L. Davies, Niels J. de Winter, L. M. E. Percival, Urs Schaltegger, David De Vleeschouwer

**Affiliations:** 1 Sedimentary Petrology Laboratory, Liège University, Allée du Six Août, 12, 4000 Liège, Belgium; 2 Department of Earth Sciences, Mountjoy Site, Durham University, South Road, Durham, DH1 3LE UK; 3 Analytical, Environmental and Geo-Chemistry (AMGC), Vrije Universiteit Brussel, Pleinlaan 2, 1050 Brussels, Belgium; 4 Département des sciences de la terre et de l’atmosphere, Université du Québec à Montréal, Montréal, Canada; 5 Department of Earth Sciences, Faculty of Geosciences, Utrecht University, Princetonlaan 8a, 3584 CB Utrecht, The Netherlands; 6 Département des sciences de la terre, Université de Genève, 1205 Geneva, Switzerland; 7 MARUM—Center for Marine Environmental Sciences, University of Bremen, Leobenerstraße, 28359 Bremen, Germany

**Keywords:** Palaeoceanography, Palaeoclimate

## Abstract

The Devonian Frasnian–Famennian (F–F) boundary marks one of the five main extinction intervals of the Phanerozoic Aeon. This time was characterized by two pulses of oceanic anoxia, named the Lower and Upper Kellwasser events, during which massive marine biodiversity losses occurred. This paper presents high-resolution magnetic susceptibility, X-ray fluorescence elemental geochemistry and carbon isotope datasets obtained from the Steinbruch Schmidt F–F boundary section (Germany). These records lead to an astronomical time calibration of the environmental changes associated with the two ocean anoxia pulses. Cyclostratigraphic interpretation indicates deposition of the black argillaceous Lower and Upper Kellwasser horizons over ~ 90 and ~ 110 kyr, respectively; approximately equivalent to the duration of one short eccentricity cycle. This study confirms that the succession of events within the Upper Kellwasser event is paced by obliquity, under a low-eccentricity orbit. Hence, astronomical insolation forcing likely contributed to the expansion of ocean anoxia and other environmental perturbations associated with these two crises. The new floating chronology established for the Steinbruch Schmidt section is anchored in numerical time by means of a radio-isotopic date, obtained from a bentonite layer interbedded between the two Kellwasser horizons. After anchoring, this time scale gives a high-precision age of 371.870 ± 0.108 Ma for the F–F boundary.

## Introduction

The Devonian Period experienced several episodes of strong environmental and climate changes, including different pulses of extinction and global carbon-cycle perturbations. The Frasnian–Famennian (F–F) boundary event stands out among these Devonian environmental perturbations, due to its strong impact on marine ecosystems ^e.g.^^1^ and is considered as one of the “Big Five” major Phanerozoic mass extinctions according to a recent synthesis and reappraisal by^1^. Classically, the sedimentary expression below and at the F–F boundary, consists of two dark shale intervals, referred to as the Lower and Upper Kellwasser shales respectively (LKW, UKW, overview in^2^). These dark shales stratigraphically correspond to two distinct positive carbon-isotope (δ^13^C) excursions recorded around the globe, including at locations where black shales did not develop^3–6^. The origin of the extinction and its specific timing have been debated for a long time. Suggested causal mechanisms for the extinction include one or more extraterrestrial impacts^7,8^, large-scale volcanism^9,10^, either climate cooling^11^ or warming^12^, and enhanced continental weathering, through mountain building^13^ or development of terrestrial forests^5,14^. Increase continental weathering in particular would have resulted in eutrophication, planktonic algal blooming and subsequent widespread marine anoxia.

**Figure 1:**
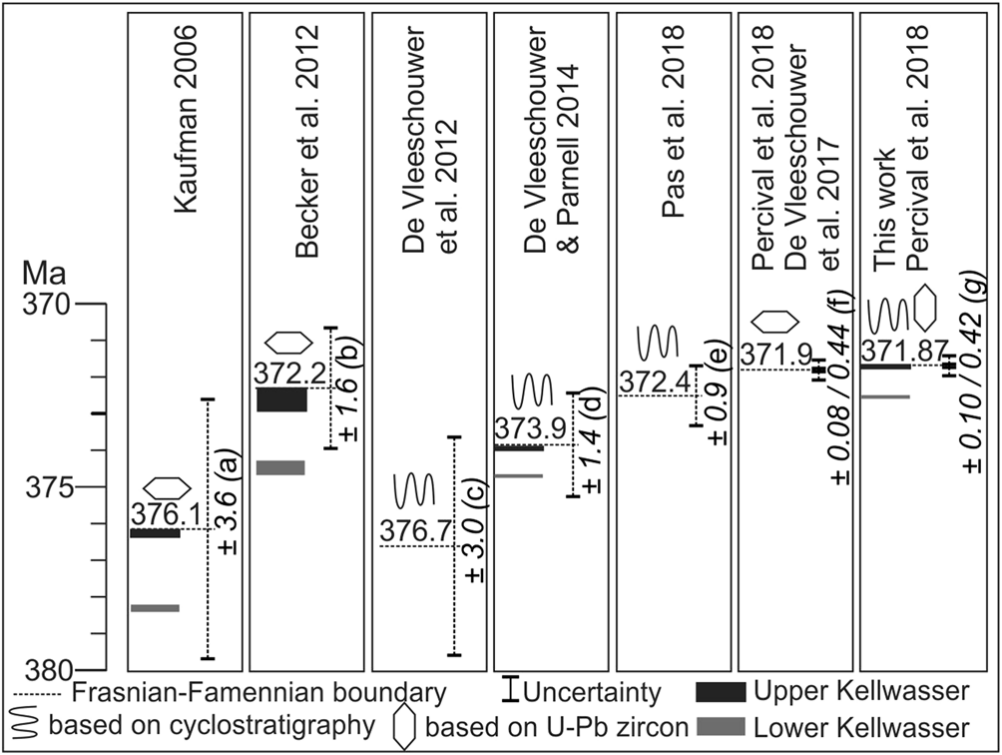
Frasnian–Famennian time scales and their uncertainties proposed in recent studies. (**a**) Uncertainty based on the 2σ error of each individual ash bed with an additional uncertainty of 2 Ma and error channel propagation age. (**b**) Uncertainty based on smoothing spline procedure with a smoothing factor of 0.45^15^. (**c**) Uncertainty based on a counting error of 1 long eccentricity cycle to the existing error bar of^16^. (**d**) Uncertainty based on Baysian age-depth Bchron model^53^. (**e**) Uncertainty includes uncertainty on the stratigraphic position of the F–F boundary, transferred in time through estimated sedimentation rate, plus one long eccentricity cycle, plus the uncertainty on the Devonian-Carboniferous boundary^22^. This uncertainty may be under evaluated, since it is based on an extrapolation from an ash beds 13.5 Myr younger than the F–F boundary. (**f**) Uncertainty based on the ash bed^25^ (372.360 ± 0.053 Ma) and extrapolation astrochronology model from^6^. (**g**) Uncertainty based on the uncertainty from the ash bed of^25^ and uncertainties obtained by comparing the different astrochronologic estimates in this paper.

Better constraints on the rate and timing of environmental change before, during and after the extinction could resolve the diverging hypotheses. Indeed, numerous different chronologies have been constructed for the F–F extinction event (synthesis in Fig. 1). Duration estimates of the interval between the onset of the first anoxic event (LKW) and the F–F boundary range between 800 and ~ 2,500 kyr, as presented below:

1. The ~ 2,500 kyr estimate^15,16^ is primarily based on three ash beds dated by U–Pb as middle Frasnian to late Famennian in age. These are the Belpre Ash [USA, 379.5 ± 1.17 Ma in the Geological Time Scale (GTS) 2012^17^, originally published by^18^], the bentonite from the Steinbruch Schmidt section [Germany, 373.68 ± 1.49 Ma in GTS 2012^17^, originally published by^19^], and a bentonite in the Piskahegan Group (Canada, 364.08 ± 2.2 Ma in GTS2012^17^, originally published by^18^). The large error bars on each of these individual radio-isotopic dates and also the use of non-chemically abraded U–Pb ages leads to substantial uncertainty, and potential inaccuracy on this ~ 2,500 kyr duration estimate.
2. Recently, strong efforts have been made to improve the Devonian time scale through cyclostratigraphy^20–22^. For the F–F interval, De Vleeschouwer et al.^23^ worked on the expression of Milankovitch cycles in the Kowala section in Poland and suggested a ~ 800 kyr time-gap between the onsets of the LKW and the UKW and a ~ 400 kyr duration for low oxygen levels during the UKW. Subsequently, De Vleeschouwer et al.^6^ complemented the Kowala section, with sections from Canada, U.S.A., China and Belgium compiled into a global cyclostratigraphic framework that places the onset of the positive LKW δ^13^C excursion ~ 800 kyr before the F–F boundary.
3. Pas et al.^22^ proposed a cyclostratigraphic framework for the whole Famennian through different cores from the Illinois Basin, anchored to a precise Famennian–Tournaisian boundary age based on U–Pb dating of uppermost Famennian bentonites from Poland^24^. This cyclostratigraphic approach resulted in a F–F boundary age of 372.4 Ma ± 0.9 Myr. No duration is proposed for the interval between the LKW and UKW. The F–F age is based on a cyclostratigraphic extrapolation from ash beds 13.5 Myr younger than the F–F boundary.
4. Recently, Percival et al.^25^ re-dated zircons from the Steinbruch Schmidt ash bed located between the LKW and UKW (utilizing modern chemical abrasion isotope dilution thermal ionization mass spectrometry (CA-ID-TIMS) U–Pb geochronology techniques as well as the Earthtime spike solutions), generating a new age of 372.360 ± 0.053 Ma for the bentonite. Using this age, extrapolating the De Vleeschouwer et al.^6^ cyclostratigraphic framework and assuming a constant sedimentation rate for the limestones deposited between the two Kellwasser horizons the F–F boundary age was estimated to be between 371.93 and 371.78 Ma^25^.

The present study brings together the latest developments in the fields of astrochronology and radio-isotopic dating to conduct a cyclostratigraphic analysis of a multi-proxy data set for the Steinbruch Schmidt section. It subsequently integrates the high-precision radio-isotopic date^25^ to tie the floating chronology to an absolute time frame. These results are then compared with previous chronologies. This approach leads to a unique high precision chronology for the F–F interval, anchored in absolute time and at unprecedented resolution.

## Geological setting, data and methods

**Figure 2:**
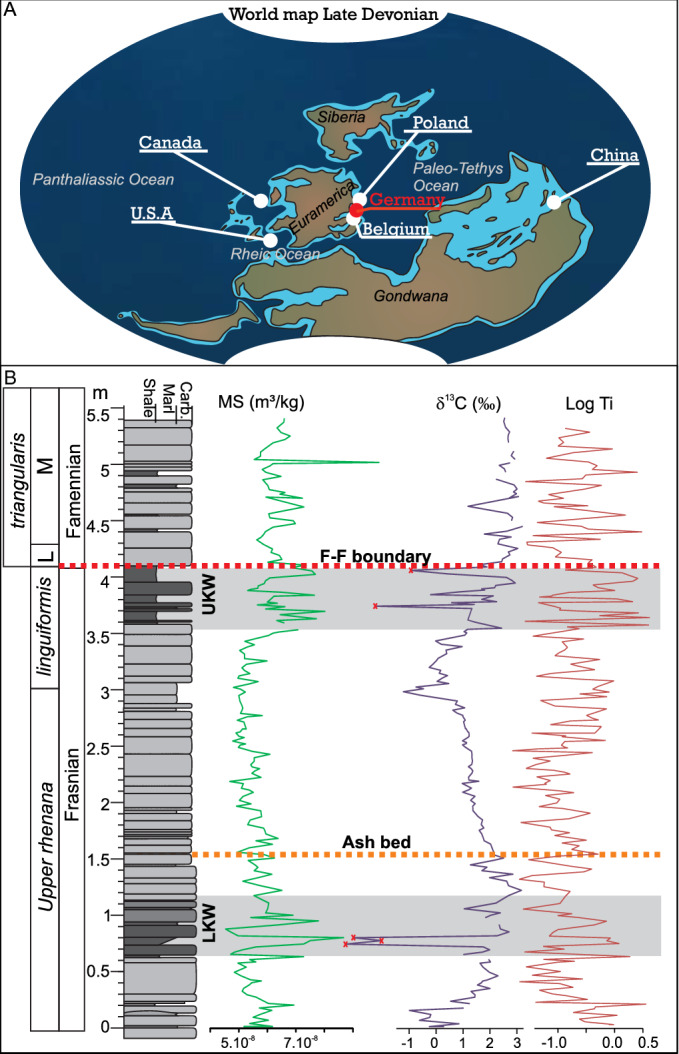
Geological Setting and lithological column of the Steinbruch Schmidt section and of other records used in this paper. (**A**) World map of Late Devonian, modified after^54^, with location (in red) of the Steinbruch Schmidt section (Germany) and other records for comparison (in grey): Fuhe (China), Kowala (Poland), Sinsin (Belgium), Section C (Western Canada), and CG-1 and H-32 from Iowa (U.S.A.). (**B**) Lithological column from Steinbruch Schmidt section, modified from^27^ and conodont biostratigraphy from^28^, with magnetic susceptibility (MS), carbon isotopes (δ^13^C) and logTi depth-series. In the δ^13^C record, outliers with the extreme low values, marked with red crosses, are considered as diagenetically altered and were removed for spectral analysis. The Frasnian–Famennian (F–F) boundary is marked by a dotted red line, the ash bed dated by^25^ by dotted orange line, and the lower and upper Kellwasser (LWK, UKW) black shale intervals are underlined by grey areas.

The Steinbruch Schmidt section was deposited at tropical palaeolatitudes (Fig. 2A) on a submarine rise, below the wave action zone, in a quiet and poorly oxygenated setting with the LKW and UKW corresponding to the deepest settings preserved^26^. This section offers outstanding conditions for a stratigraphic framework and timescale that can be globally correlated. It contains well-developed Lower and Upper Kellwasser black-shale intervals and associated positive carbon-isotope excursions^3,27^, a high-precision radio-isotopic date^19,25^, as well as an excellent biostratigraphic framework^28^. For this study, the 5.3 m of outcropping section, which ranges from 65 cm below the LKW to 75 cm above the UKW (Fig. 2B, N = 264) was sampled at high-resolution (2 cm) and each sample was measured for magnetic susceptibility, carbon isotopes and XRF geochemistry, following the protocol below. As mentioned above, there is an ash bed in the Steinbruch Schmidt section, intercalated between the two Kellwasser horizons. The “Bed 36” ash occurs 1.5 m higher than the base of our sampling interval and 2.5 m below the F–F boundary, and was recently re-dated by^25^ to 372.360 ± 0.053 Ma.

Magnetic susceptibility was measured 3 times on each sample, with a KLY-3 (AGICO Kappabridge) at Liège University (Belgium), averaged and weighted to produce mass-specific (m^3^/kg) data. At the Vrije Universiteit Brussel (Belgium), a Bruker M4 Tornado, operated under near-vacuum conditions (20 mbar) was used for the μXRF measurements. The conditions used were: a 30 W Rh anode metal-ceramic X-ray tube and a 30 mm^2^ SSD with a resolution of 145 eV (Mn–Ka). The X-ray beam was focused by a poly-capillary lens on a spot with a diameter of 25 mm (Mo–Ka). An integration time of 60 s per point was chosen, satisfying the conditions for reaching the Time of Stable Accuracy and Time of Stable Reproducibility and providing the ideal compromise between high analytical accuracy and precision and high sample throughput (^29^ for analytical details). The carbon (δ^13^C) stable isotope ratios of bulk powered carbonate material were also determined at the Vrije Universiteit Brussel, using a NuPerspective isotope ratio mass spectrometer (IRMS) interfaced with a NuCarb automated carbonate device. Acidification of the samples took place at a temperature of 70 °C. All values are expressed relative to the Vienna Pee Dee Belemnite (‰VPDB) standard. Calibration was carried out using an in-house Marbella limestone (MAR) standard (+ 3.41 ‰VPDB) calibrated against the international NBS-19 standard^30^. On the basis of replicated measurements of the MAR standard, reproducibility errors on δ^13^C are < 0.05 ‰ (1 σ).

All time-series analyses are performed on the R platform^31^. All proxy series are interpolated to 0.02 m. First, the function eTimeOpt within the *Astrochron* package^32^ is used to evaluate sedimentation rates and their stratigraphic evolution^33,34^. The eTimeOpt method provides a quantitative assessment of a sequence of possible sedimentation rates, adopting a sliding window approach. Within each window, sedimentation rates that result in precession amplitude modulation patterns and frequency ratios in accord with astronomical theory are singled out. The eTimeOpt function requires the user to provide target eccentricity and precession periodicities to be tested. For the Devonian, this requires non-trivial assumptions, as the periodicities of obliquity and precession in “deep time” are affected by the tidal dissipation of energy. The precession periodicities (16.85, 19.95 kyr) as calculated by^35^ are selected. Long and short eccentricity relate to the shape of Earth’s orbit and not to its rotational speed, hence eccentricity is not affected by tidal dissipation. Therefore, the 100 kyr and 405 kyr periodicities have remained unchanged throughout geological history. Numerical calculations by Laskar et al.^36,37^ have demonstrated the extraordinary stability of the periodicity of the 405 kyr term, often referred to as the metronome of the Phanerozoic. In this study, eccentricity periodicities of 94.9, 98.9, 123.8, 130.7 and 405 kyr are adopted^37^.

The eTimeOpt chronology is then compared with a time scale obtained though the age modelling protocol designed by^6^. These authors correlated 6 globally-distributed Frasnian–Famennian sections (Poland, Kowala section; western Canada, Section C; Iowa, H-32 and CG-1 drill core; Belgium, Sinsin section; and South China, Fuhe section, Fig. 2A), through tie-points obtained by visually correlating distinct features in the stratigraphic trends of magnetic susceptibility and carbon-isotope compositions, whilst respecting conodont biostratigraphy constraints. A tentative relative age was assigned to each of those tie-points, abiding to the 405 kyr astrochronologic framework for Western Canada (Fig. 7 in^6^). Those authors then applied a Monte Carlo procedure that distorts time-differences between consecutive tie-points, i.e. small-scale stretching and squeezing between tie points. Their goal was to reach the best expression of the Milankovitch cycles, under the presumption that the chronology with the clearest orbital signature advocated to be the most accurate (detailed description in^6^). This technique is called the Optimized Relative Timing Approach (ORTA). The new results from the Steinbruch Schmidt section are incorporated into this correlation framework and assigned relative age tie-points (with 0 kyr corresponding to the F–F boundary) identical to those in^6^.

Finally, the Wavelet Transform evolutive spectra (CWT^38^), obtained through the biwavelet package^39^ is applied. Continuous wavelet is an evolutive spectral techniques that reveals the evolution of periodicities for the studied sections and detects changes in cycle thickness and, hence, sediment accumulation rate. These CWT results are compared with the results from eTimeOpt and ORTA.

## Results

Magnetic susceptibility, logTi and δ^13^C are selected as proxies for the section (Fig. 2B). Magnetic susceptibility (MS) is viewed as a proxy for detrital input e.g.^40^, logTi to reflect the relative rate of siliciclastic sedimentation^41,42^, and carbon-isotope variations as indications for net changes in the carbon cycling between the ocean, atmosphere, biosphere and lithosphere^43^. Ti evolution includes very strong peaks. By using the logarithmic signal, modest base-level variations are enhanced while positive peak values are reduced. The Steinbruch Schmidt section is dominated by carbonate lithologies, with the two Kellwasser dark marls or shales. Within these argillaceous intervals, MS increases. Although the LKW dark marly layer ranges between 0.6 and 1.15 m, the carbon isotope ratios start to increase at 0.15 m, reach a peak around 1.2 m and then decreases until about 3 m. The UKW marly dark layer ranges between 3.6 and 4.1 m with the carbon isotope ratios increasing from 3 m. LogTi values display strong variations, with a slight increase in the average value during the marly Kellwasser intervals.

**Figure 3:**
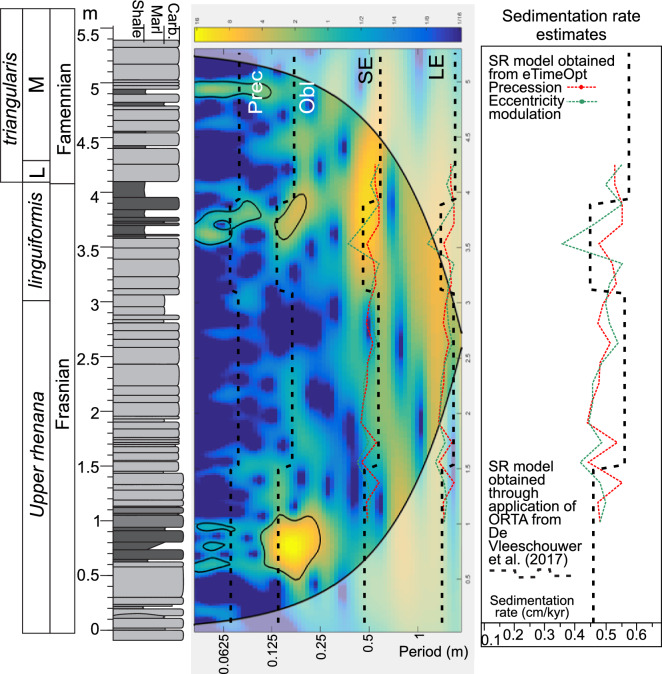
Continuous Wavelet Transform of the Magnetic susceptibility signal, together with the different results from eTimeOpt (red and green lines) and ORTA (black dotted line) on the magnetic susceptibility record. *LE* long eccentricity, *SE* short eccentricity, *Obl* obliquity, *Prec* precession.

The application of eTimeOpt produces a first assessment of a potential Milankovitch imprint in these different proxy records. As an input parameter, eTimeOpt requires an estimate of a range of plausible sedimentation rates for the studied interval. As outlined in the introduction, LKW-to-F–F-boundary duration estimates range from 800 kyr^6^ to more than three times that timespan^15^. The corresponding stratigraphic interval at the Steinbruch Schmidt section consists of about 400 cm of section. Hence, plausible sedimentation rates range between 0.17 and 0.5 cm/kyr. The eTimeOpt approach is applied to these different records (logTi, MS and δ^13^C) with a grid of 100 sedimentation rates between 0.15 and 0.55 cm/kyr range. The results of eTimOpt for amplitude modulation of precession, give evolving sedimentation rates (for LogTi, MS and δ^13^C) ranging in average values between 0.47 and 0.53 cm/kyr (Table 1, Fig. 3, Fig. Suppl. Mat. 1).

**Table 1:** Results obtained by different spectral analysis techniques on different proxies on the Steinbruch Schmidt section.

Spectral techniques	Record	Sed rate boundaries (cm/kyr)	Duration of whole record (kyr)	Sed rate (cm/kyr)	Duration of interval between LKW and F-F (kyr)	Difference with median (kyr)	Difference with ORTA MS (kyr)
eTimeOpt_Prec_SE	MS	0.1–0.55	1,062	0.50	694.0	11.0	16.0
eTimeOpt_SE_LE	MS	0.1–0.55	1,085	0.49	697.5	12.0	39.0
eTimeOpt_Prec_SE	LogTi	0.1–0.55	1,007	0.53	716.0	66.0	39.0
eTimeOpt_SE_LE	LogTi	0.1–0.55	1,124	0.47	812.0	51.0	78.0
eTimeOpt_Prec_SE	δ^13^C	0.1–0.55	1,129	0.47	708.0	56.0	83.0
eTimeOpt_SE_LE	δ^13^C	0.1–0.55	1,100	0.48	743.0	27.0	54.0
ORTA	MS		1,046	0.51	775.0	27.0	0.0
ORTA	LogTi		1,073	0.49	843.0	0.0	27.0
ORTA	δ^13^C		994	0.53	809.0	79.0	52.0

			Median	Median	Median	Average	Average
			1,073.0	0.49	743.00	36.6	43.1
		Standard deviation	47.31	0.02	56.14		

Spectral techniques include eTimeOpt_Prec_SE, corresponding to the eTimeOpt technique focussing on the modulation of precession by short eccentricity; eTimeOpt_SE_LE, corresponding to the eTimeOpt technique focussing on the modulation of short eccentricity by long eccentricity and ORTA Optimized Relative Timing Approach. Records includes magnetic susceptibility (MS), Log Ti and Carbon isotopes (δ^13^C). Results are expressed as the duration of the whole record in kyr, sedimentation rate (SedRate in cm/kyr), duration of the interval between the onset of the Lower Kellwasser (LKW) Carbon isotopic excursion and Frasnian–Famennian (F–F) boundary; and difference with median value.

These different eTimeOpt assessments provide total duration estimates for the studied interval (Table 1) of 1,129 kyr (δ^13^C), 1,062 kyr (MS) and 1,007 kyr (logTi). However, eTimeOpt for amplitude modulation of precession cannot consider the full sedimentation rate interval as the proxy-series resolution is too low to resolve precession cycles for sedimentation rates lower than 0.26 cm/kyr. Running eTimeOpt again on all proxies, investigating the amplitude modulation of short eccentricity by long eccentricity circumvents this problem. These settings lead to eTimeOpt results for the full sedimentation rate range. Despite the fundamentally different eTimeOpt settings, the sedimentation rates remain similar (ranging between 0.47 to 0.49 cm/kyr, Table 1, Fig. 3, Fig. Suppl. Mat. 1), which translate into duration estimates of 1,100 kyr (δ^13^C), 1,085 kyr (MS) and 1,124 kyr (logTi) for the whole section (Table 1). The eTimeOpt results appear thus consistent, for both amplitude modulation of precession by short eccentricity or short eccentricity by long eccentricity. Furthermore, the eTimeOpt results point to a relatively short duration between the base of the LKW δ^13^C excursion and the F–F boundary, ranging between 694 and 812 kyr, which confirms the rather short LKW-to-F–F-boundary duration estimates by^6^.

**Figure 4:**
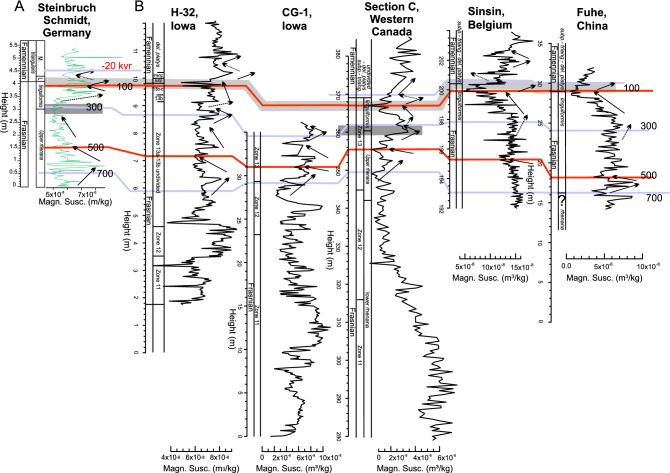
Magnetic susceptibility correlation of Steinbruch Schmidt section with other F–F records (H-32, CG-1, Section C, Sinsin, and Fuhe, see^6^) and visual distinct magnetic susceptibility features for correlations (red lines and arrows). These tie points have to respect the biostratigraphic constraints (underlined by grey area) and carbon isotope correlations (Fig. 5, blue lines). The black numbers are ages assigned to each tie-point, according to the existing astrochronologic framework of Section C and Kowala^23,55^. *Fam.* Famennian, *h.-j.* hassi-jamieae, *U.* upper, *linguif.* linguiformis, *subp.* subperlobata, *triang.* triangularis, *del.* platys delicatula platys.

**Figure 5:**
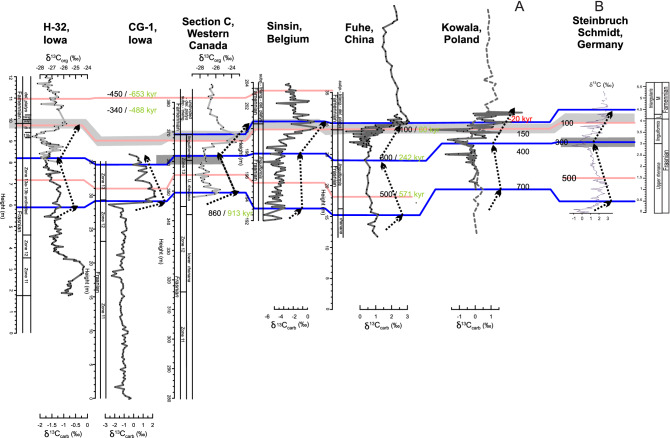
Carbon isotope correlation of Steinbruch Schmidt section with other F–F records (H-32, CG-1, Section C, Sinsin, Fuhe, and Kowala, see^6^) obtained by visually correlating distinct features in carbon isotope geochemistry (blue ties and dotted arrows). These tie points are accepted only if they respect the biostratigraphic constraints (underlined by grey area) and magnetic susceptibility correlations (Fig. 4, red lines). The black numbers are ages assigned to each tie-point, according to the existing astrochronologic framework of Section C and Kowala^23,55^. Abbreviations as in Fig. 4.

Next, Steinbruch Schmidt proxy records are placed within the ORTA framework developed by De Vleeschouwer et al.^6^ A correlation between the Steinbruch Schmidt record and the six records from^6^ is established by identifying five correlation tie-points based on biostratigraphy, δ^13^C chemostratigraphy, and magnetic susceptibility proxy records (Figs. 4, 5). The decrease in the amount of tie points (from 7 to 5) compared to^6^ gives more freedom for distortions. The five tie-points are assigned tentative relative ages with respect to the F–F boundary of − 20 kyr (above the boundary), 100 kyr, 300 kyr, 500 kyr and 700 kyr (below the boundary, Figs. 4, 5), as prescribed for the ORTA procedure. The 720 kyr that separate the extreme tie points dictate a mean sedimentation rate of 0.55 cm/kyr, only slightly higher than the sedimentation rates obtained through eTimeOpt. The ORTA transforms the signal from the distance to the time domain between the different tie-points using a Monte Carlo approach, retaining the chronology that has the best expression of the expected Milankovitch frequencies between all different proxies. The algorithm yields an optimized result with a total duration of 1,045 ka, corresponding to a mean sedimentation rate of 0.51 cm/kyr for magnetic susceptibility, as well as 1,073 kyr and 0.49 cm/kyr for LogTi and 1,046 and 0.49 cm/kyr (Table 1). This result fall close to those obtained through eTimeOpt. Finally, the results are examined using a continuous wavelet transform (Fig. 3, Fig. Suppl. Mat. 1), which shows strong spectral power at periods of 0.4–0.7 and 1.5–2 m. These periods fit respectively with short and long eccentricity, with sedimentation rates around 0.5 cm/kyr; they fully agree with the results from eTimeOpt and ORTA (Table 1).

Two fully independent spectral techniques (eTimeOpt from^33^, and the ORTA from^6^) applied on the different proxies from the Steinbruch Schmidt section, lead to similar results for the duration of the whole section. These duration estimates for the section range between a minimum of 994 kyr to a maximum value of 1,129 kyr (Table 1), whilst duration of the interval between the beginning of the δ^13^C excursion and the F–F boundary ranges between 694 and 843 kyr. The narrow range of results is also illustrated by the average difference between different duration estimates and the median estimate of only 37 kyr (Table 1).

## Discussion

The subsequent discussion is primarily based on the results obtained by ORTA. The focus lies on ORTA from the magnetic susceptibility result for the following reasons (Fig. 3, Fig. Suppl. Mat. 1): (1) eTimeOpt is an evolutive technique that works with sliding windows. Results are reported for window mid-points, which implies that the results for the extremities of the record need to be extrapolated. (2) For the lower and upper Kellwasser black shales, a rather low sedimentation rate is expected^44^, which is indeed the case with ORTA on Magnetic susceptibility, but not with eTimeOpt or on ORTA on other proxies. (3) The results from ORTA exhibit a slightly better fit with the band of strong power in the continuous wavelet transform and evolutive harmonic analysis. Nevertheless, all results are very close as the difference between the various results obtained through the different techniques and those from ORTA on magnetic susceptibility never amounts to more than 83 kyr (Table 1). The Supplementary Materials shows other results from eTimeOpt and from ORTA on all proxies in details (Figs. Suppl. Mat. 1–3), but the outcomes are similar and the slight differences between the models will be discussed and integrated.

**Figure 6:**
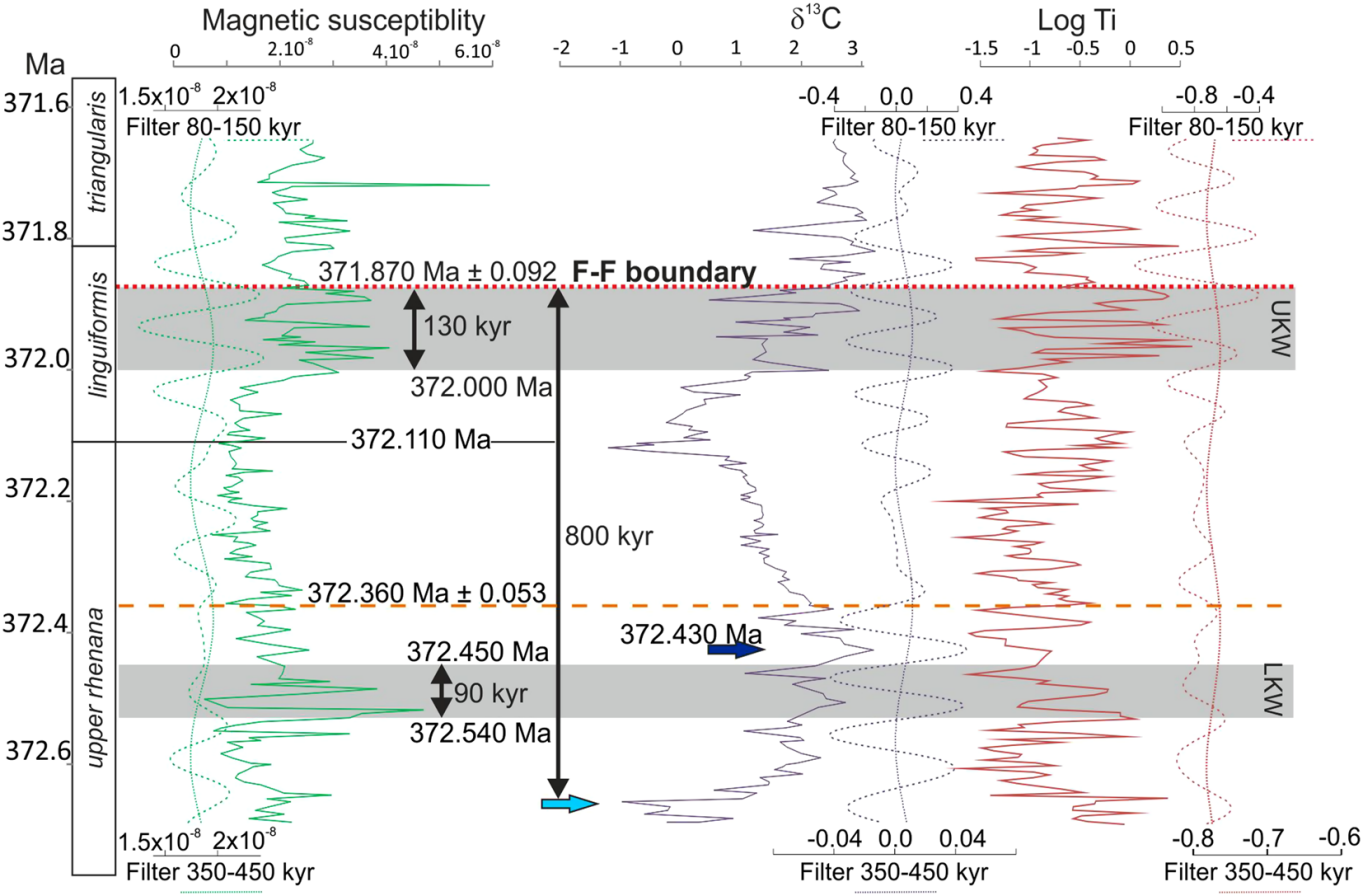
Integrated anchored chronology for the Frasnian–Famennian boundary, obtained through the combination of ORTA and the^25^ radio-isotopic date (orange dotted line). The grey bands mark the extension of the lower and upper black shally Kellwasser intervals. The light blue arrow marks the onset of positive carbon excursion associated with the Lower Kellwasser (372.670 ± 0.102 Ma), the dark blue arrow marks the maximum of the carbon excursion associated with the Lower Kellwasser (372.430 ± 0.102 Ma). The red dotted line marks the Frasnian–Famennian (F–F) boundary (381.870 ± 0.092 Ma).

The Steinbruch Schmidt bentonite layer at bed 36, located between the LKW and the UKW horizons has been recently re-dated by^25^, who reported a weighted mean age of 372.360 ± 0.053 Ma. Combining this age with the present cyclostratigraphy provides an integrated time scale for the Frasnian–Famennian boundary interval (Fig. 6) where the age of the F–F boundary falls at 371.87 Ma, considering the 5 short eccentricity cycles between the Bed 36 bentonite and the F–F boundary. The new F–F boundary age is younger than most previous estimates (see Fig. 1). However, it is important to note that this young age is primarily inherited from the younger U–Pb age^25^, rather than resulting from issues in the cyclostratigraphic framework, which conforms with previous astrochronologic studies^6^. The Lower and Upper Kellwasser black argillaceous horizons represent respectively between 80 to 96 kyr and 100 to 130 kyr of time (equivalent to approximately one short eccentricity cycle, Fig. 6, Suppl. Mat. 2). The different proxies (MS, LogTi, δ^13^C) are all in phase (Fig. 6). The interval between the onset of the Lower Kellwasser carbon-isotope excursion (372.6*7* Ma) and the Frasnian termination is between 690 and 843 kyr *(*Fig. 6, Table 1, Fig. Suppl. Mat. 2).

Uncertainty assessment on cyclostratigraphic results is a challenging endeavour^45^. An uncertainty assessment can be proposed by considering the results obtained through different cyclostratigraphy techniques and proxies, associated with the uncertainty on the ash bed. Considering the techniques applied on different proxies generates seven different cyclostratigraphic duration estimates for the studied stratigraphic interval (Table 1). The standard deviation (σ) between these techniques is 47 kyr. Since the ash bed and the cyclostratigraphic estimates are independent, the square root law combines these uncertainties (as 2σ, 2 × sqrt(σ1^2^ + σ2^2^)). Percival et al.^25^ proposed different uncertainties, one 2σ at ± 53 kyr, which represents only the measurement uncertainty and allows comparison with ash bed dates obtained through the same technique and one 2σ at ± 410 kyr, which includes the measurement, tracer and also decay constant uncertainties and must be employed when making comparisons between U–Pb ages and argon-argon (Ar–Ar) ages. Consideration of those uncertainties gives a Frasnian–Famennian boundary age of 371.870 ± 0.108 Ma for the uncertainty to be compared with other U–Pb ages and 371.870 ± 0.420 Ma to be compared with Ar–Ar ages.

The newly constructed integrated and anchored time scale obtained for the Steinbruch Schmidt section also allows for the evaluation of the impact of astronomical forcing on the Kellwasser environmental perturbations. A connection between orbital forcing and organic carbon accumulation has been frequently postulated^46–48^. However, two contrasting hypotheses exist regarding the exact nature of this link (synthesis in^49^): one in which organic matter accumulation is favoured during eccentricity maxima and one during eccentricity minima. The eccentricity maxima hypothesis involves a context of strong seasonal contrast between dry and wet seasons, allowing strong fluvial discharge, productivity blooms and organic-matter accumulation. This model is classically invoked to explain high-productivity dark sapropel layers from the Pliocene of the Mediterranean region^50,51^. The eccentricity minima hypothesis invokes a scenario of enhanced preservation, involves low seasonal contrast, associated with stable conditions, with water mass stratification allowing organic matter preservation and persistent anoxia. This model is used to explain the Cretaceous Oceanic Anoxic Events^47,48,52^.

De Vleeschouwer et al.^6^ reported maximum obliquity power in the δ^13^C proxy starting a few tens of thousands of years before the F–F boundary. They identified that the obliquity signal is strong in this interval because it represents a 2.4 Myr minimum in eccentricity. In such an astronomical configuration, the amplitude of precession is muted, and obliquity is the dominant astronomical parameter that directly influences the distribution of insolation over the globe and over the seasons. This scenario is similar to that proposed for Cretaceous Ocean Anoxic Event 2 during the Cenomanian–Turonian interval^47,52^ and fits with the eccentricity minima enhanced preservation model. The Steinbruch Schmidt section δ^13^C proxy series tests this hypothesis. The δ^13^C signal is long enough to includes sufficient amount of short eccentricity to focus on the evolution of obliquity and eccentricity power, to confirm or refute the minima eccentricity hypothesis.

**Figure 7:**
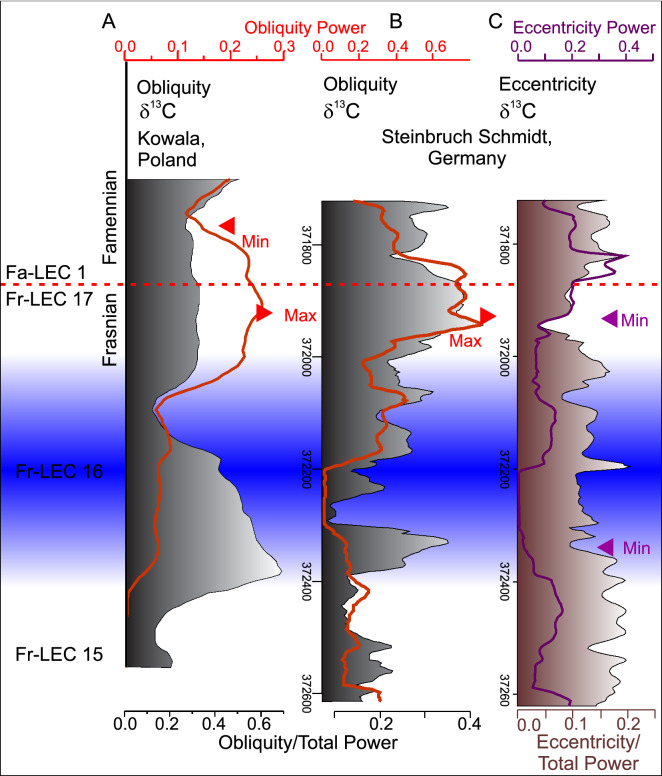
Changing imprint of obliquity and eccentricity forcing across lower and upper Kellwasser time scale. All analyses have been conducted using a 3–2π MTM power spectra and a 150 kyr moving window. (**A**) Obliquity power (red line) and obliquity/total power (black and grey shaded area) of the δ^13^C record at Kowala section in Poland, by^6^. (**B**) Obliquity power (1/21 to 1/40, red line) and obliquity/total power (black and grey shaded area) of the δ^13^C record at Steinbruch Schmidt. (**C**) Eccentricity (1/80 to 1/150, purple line) and eccentricity/total power (brown shaded area) of the δ^13^C record at Steinbruch Schmidt. Comparison with Frasnian long eccentricity cycles (Fr-LEC) defined in^6^, such as Fr-LEC 15–17 (Frasnian long Eccentricity cycle 15–17) and Fa-LEC 1 (Famennian long eccentricity cycle 1).

The obliquity power of the tuned (by eTimeOpt and by ORTA) δ^13^C record at Steinbruch Schmidt documents similar trends to those of De Vleeschouwer et al.^6^, with an obliquity power reaching maximum values slightly below the F–F boundary (Fig. 7, Fig. Suppl. Mat. 3). At the same stratigraphic level, eccentricity power reaches a minimum, confirming that the two astronomical controls are indeed inversely proportional, as expected from astronomical theory. In analogy with the Cretaceous Oceanic Anoxic Events, the Steinbruch Schmidt results support an Upper Kellwasser global carbon-cycle perturbation generated by eccentricity minima model.

To summarise, both Kellwasser events occur over approximately only a single short eccentricity cycle and are initiated during the early (low-eccentricity) phase of a 405 kyr cycle. An extreme eccentricity minimum, during which a 2.4 Myr and 405 kyr minima culminated, likely initiated the Upper Kellwasser Event. This eccentricity minimum 2.4 Myr configuration potentially explains why the Upper Kellwasser appears to have been more severe than the lower Kellwasser. Such low-eccentricity configuration decreases seasonality and reduces circulation and overturns ocean basins, facilitating the accumulation of organic matter^6,47^.

## Conclusions

For the first time, an astrochronology is compiled for a Devonian section that also comprises a high-precision radio-isotopic date. This unique integration of chrono- and cyclostratigraphy results in a precisely time-calibrated sequence of events across the F–F boundary, well-anchored in numerical time.

The Steinbruch Schmidt section (Kellerwald, Germany) is ideal for this combined cyclostratrigraphy/absolute age approach because it is characterized by the rare combination of well-expressed dark shale Kellwasser intervals, reliable environmental proxies and a datable bentonite ash. The carbon isotopes, logTi and magnetic susceptibility series of the section provide new insight in the astronomical signature of environmental change across the F–F boundary and build a cyclostratigraphy for the transition. Both Kellwasser anoxic episodes are interpreted to have lasted for about one short eccentricity cycle and to have begun during a 405 kyr eccentricity minima. The Upper Kellwasser appears more severe in its expression as it corresponds to a 2.4 Myr minimum in eccentricity (compared to the Lower Kellwasser, which occurs at a 405 kyr minimum eccentricity). This study confirms the hypothesis put forward by^6^ that obliquity, under a low-eccentricity orbit make the Earth system more prone to developing marine anoxia, helping the development of the low-oxygen Kellwasser events preceding the F–F boundary mass extinction. However, the main result of this study is a new F–F boundary age of 371.870 ± 0.108 Ma (to be compared with U–Pb ages).

This study demonstrates that even in the Palaeozoic a < 100 kyr time scale resolution is within reach when cyclostratigraphy can be combined with high-precision absolute ages. However, such chronologies can only be obtained through optimal proxy selection and multiproxy approach for robustness, careful comparison of different techniques for time-series analysis and an integrated stratigraphy approach.

## Supplementary information

Supplementary Information
