# Supplementary material for: Anchoring the Late Devonian mass extinction in absolute time by integrating climatic controls and radio-isotopic dating
Source: Sci Rep. 2020 Jul 31;10:12940. doi: 10.1038/s41598-020-69097-6 (PMC7395115; doi:10.1038/s41598-020-69097-6)
Supplement: Supplementary file 1 — Supplementary Information [file 41598_2020_69097_MOESM1_ESM.pdf]

Anchoring the Late Devonian Mass extinction in absolute time by integrating climatic controls and radio-isotopic dating.

Da Silva, A.C.[1], Sinnesael, M.[2], Claeys, Ph.[3], Davies, J.H.F.L.[4], de Winter, N.J.[3,5], Percival, L.[3], Schaltegger, U.[6], De Vleeschouwer, D.[7]

1. Liège University, Sedimentary Petrology Laboratory, Allée du Six Août, 12, Quartier Agora, 4000 Liège, Liège University, Belgium
2. Department of Earth Sciences, Mountjoy Site, Durham University, South Road, Durham DH1 3LE, UK.
3. Analytical, Environmental and Geo-Chemistry (AMGC), Vrije Universiteit Brussel, Pleinlaan 2, 1050 Brussels, Belgium.
4. Département des sciences de la Terre et de l'atmosphère, Université du Québec à Montréal, Montréal, Canada
5. Department of Earth Sciences, Faculty of Geosciences, Utrecht University, Utrecht, The Netherlands
6. Département des sciences de la Terre, Université de Genève, 1205, Genève, Switzerland.
7. MARUM—Center for Marine Environmental Sciences, University of Bremen, Leobenerstraße, 28359 Bremen, Germany.

* Corresponding authors: ac.dasilva@uliege.be and ddevleeschouwer@marum.de

Supplementary Material :

Suppl. Mat. 1. Continuous Wavelet Transform, Evolutive Harmonic Analysis of the Magnetic susceptibility signal, together with the different results from eTimeOpt (red and green lines) and the ORTA results (black dotted line) on the magnetic susceptibility, carbon isotope and LogTi records. LE = Long eccentricity, SE = short eccentricity, Obl = Obliquity, Prec = Precession.

Suppl. Mat. 2. Comparison of attached time scales for the Frasnian–Famennian boundary obtained by different techniques. (A) Conodont biostratigraphy[28] and tuned magnetic susceptibility, carbon isotope and LogTi records, obtained by ORTA (similar as Figure 6) and (B) Results of eTimeOpt modulation of precession by short eccentricity on the carbon isotope record (respective maximal total duration estimate; Tab. 1) and (C) Results of eTimeOpt modulation of precession by short eccentricity on the LogTi records (respective minimal total duration estimate; Tab. 1). The orange dotted line marks the position of the bentonite dated by[25] that was used to tie the time scale (372.36 ± 0.102 Ma). The grey bands mark the extension of the lower and upper black shally Kellwasser intervals. The light blue arrow marks the onset of positive carbon excursion associated with the Lower Kellwasser (372.67 ± 0.102 Ma), the dark blue arrow marks the maximum of the carbon excursion associated with the Lower Kellwasser (372.43 ± 0.102 Ma). The Lower and Upper Kellwasser are respectively lasting between 80 and 96 kyr and 100 and 130 kyr.

Suppl. Mat. 3. Changing imprint of obliquity and eccentricity forcing across Lower and Upper Kellwasser on the time scales obtained by ORTA and eTimeOpt. All analyses have been conducted using a 3–2π MTM power spectra and a 150-kyr moving window. A) Obliquity Power (red line) and Obliquity/Total Power (black and grey shaded area) of the δ13C record at Kowala section in Poland, by [6] ; B-E) Obliquity Power (1/21 to 1/40 kyr, red line) and Obliquity/Total Power (black and grey shaded area) and Eccentricity (1/80 to 1/150 kyr, purple line) and eccentricity/Total power (brown shaded area) of the $\delta^{13}C$ record at Steinbruch Schmidt obtained by B-C) ORTA and by D-E) eTimeOpt. Comparison with Long eccentricity cycles (LEC) defined in[6] . All results show similar maximal obliquity imprint just below the F-F boundary, corresponding to a minimal eccentricity imprint.

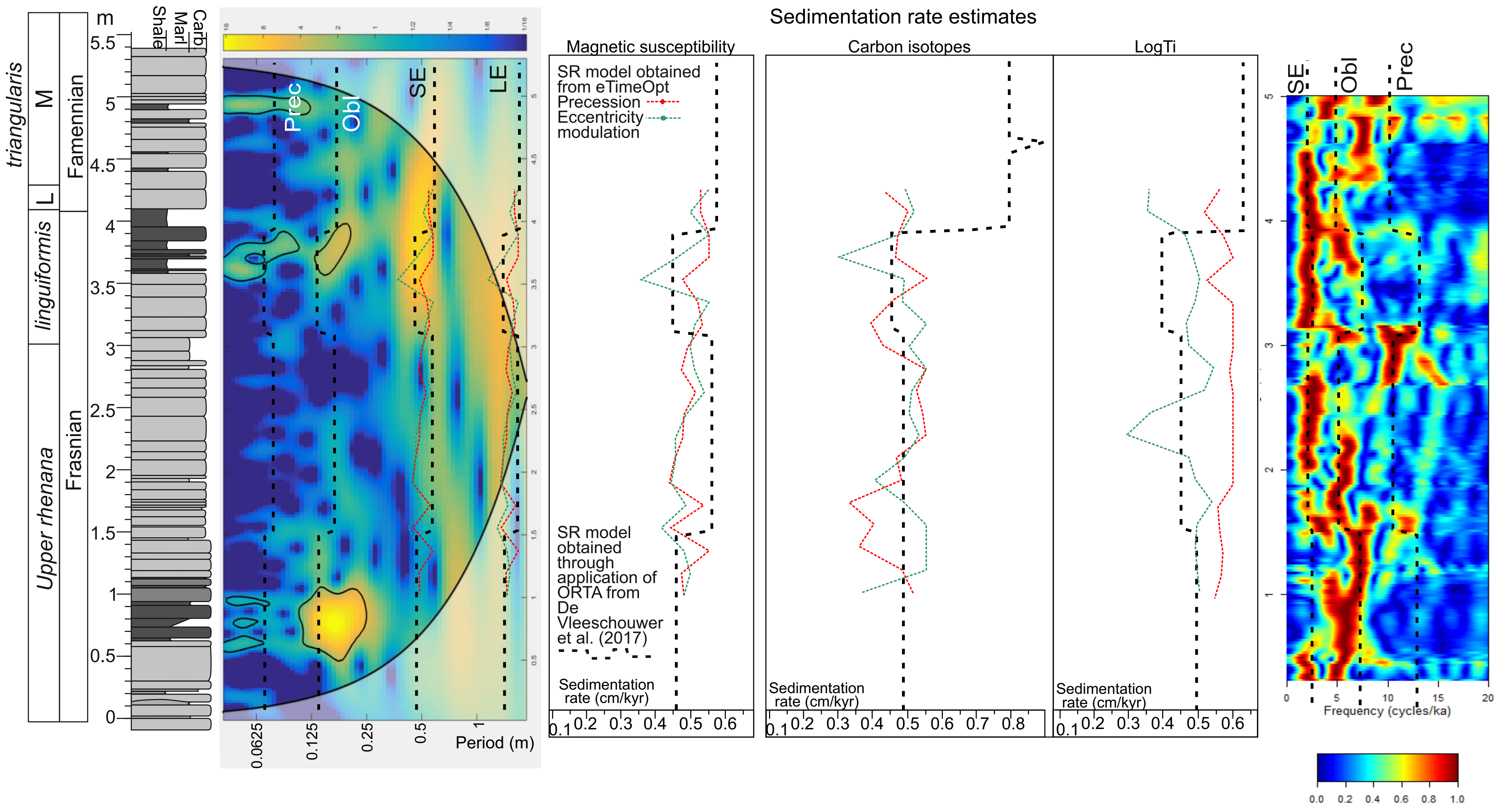

Fig. Suppl. Mat. 1

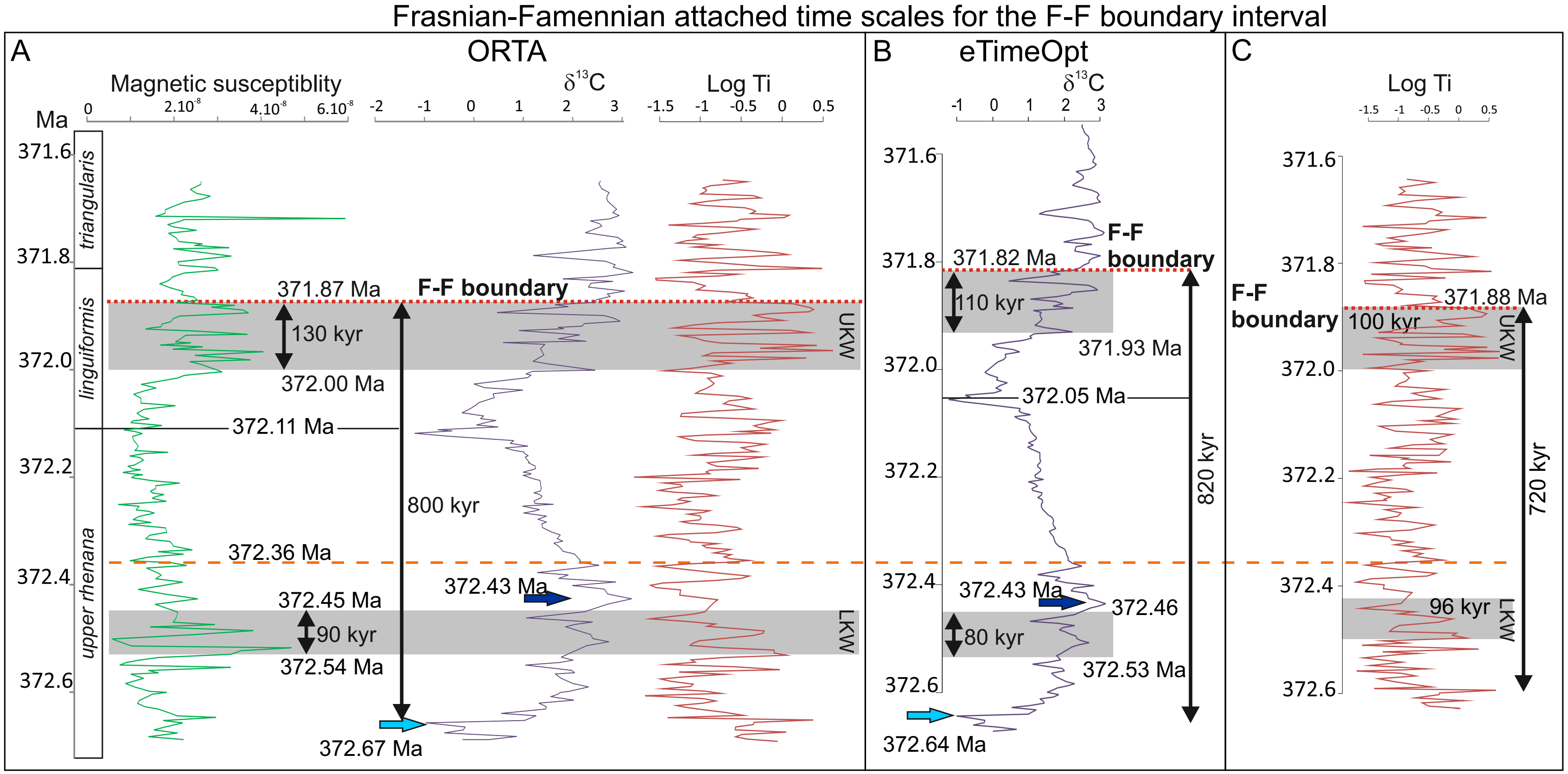

Fig. Suppl. Mat. 2

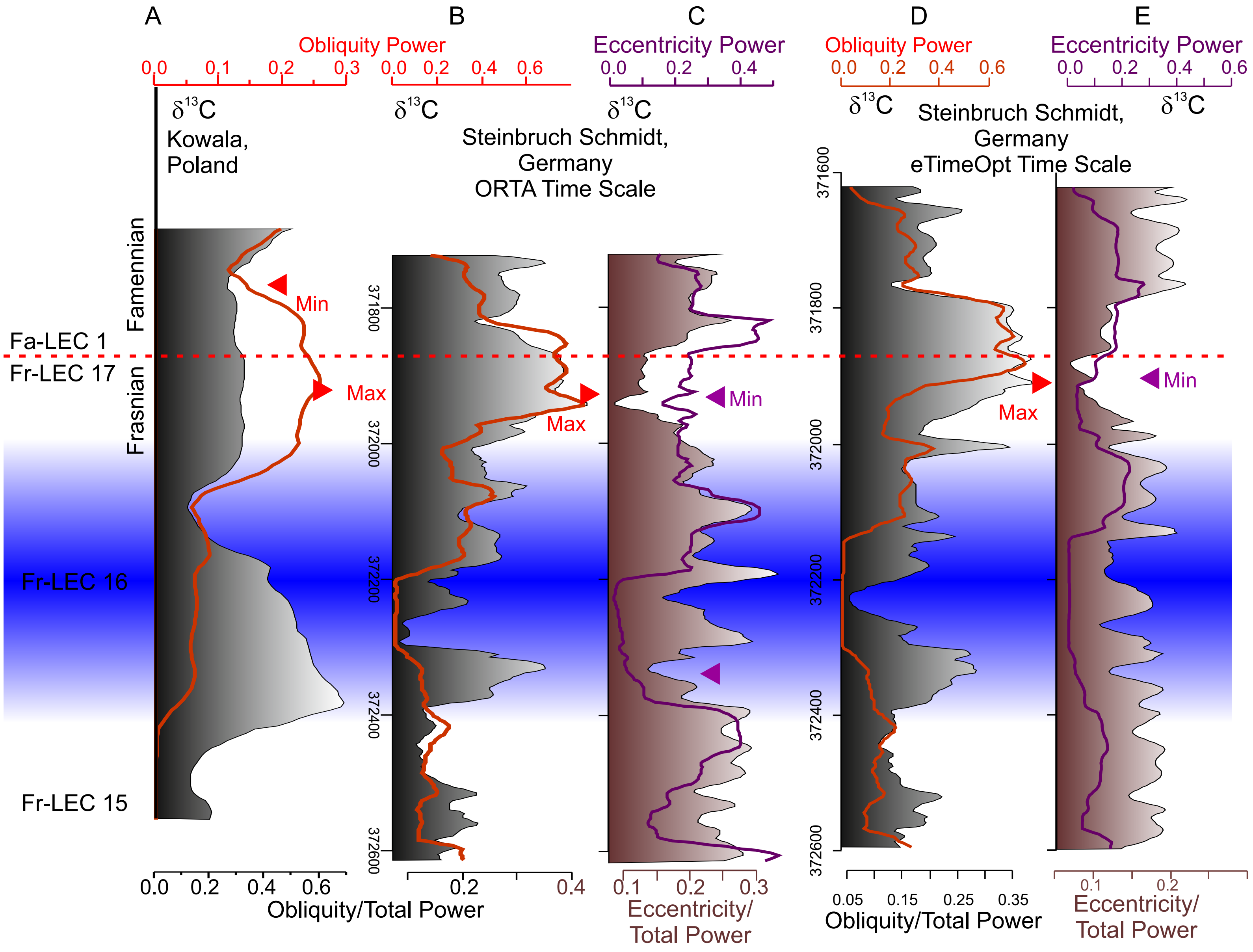

Fig. Suppl Mat 3
